# Loss of DSTYK activates Wnt/β-catenin signaling and glycolysis in lung adenocarcinoma

**DOI:** 10.1038/s41419-021-04385-1

**Published:** 2021-12-01

**Authors:** Chenxi Zhong, Ming Chen, Yu Chen, Feng Yao, Wentao Fang

**Affiliations:** 1 Department of Thoracic Surgery, Shanghai Chest Hospital, Shanghai Jiao Tong University, 200030 Shanghai, China; 2 Department of Thoracic Surgery, Shandong Zaozhuang Mining Group Central Hospital, 277800 Zaozhuang, China

**Keywords:** Cancer metabolism, Non-small-cell lung cancer, Non-small-cell lung cancer

## Abstract

Aberrant activation of Wnt/β-catenin signaling and dysregulation of metabolism have been frequently observed in lung cancer. However, the molecular mechanism by which Wnt/β-catenin signaling is regulated and the link between Wnt/β-catenin signaling and cancer metabolism are not fully understood. In this study, we showed that the loss of dual serine/threonine tyrosine protein kinase (DSTYK) led to the activation of Wnt/β-catenin signaling and upregulation of its target gene, lactate dehydrogenase (LDHA), and thus the elevation of lactate. DSTYK phosphorylated the N-terminal domain of β-catenin and inhibited Wnt/β-catenin signaling, which led to the inhibition of cell growth, colony formation and tumorigenesis in a lung adenocarcinoma mouse model. DSTYK was downregulated in lung cancer tissues, and its expression was positively correlated with the survival of patients with lung adenocarcinoma. Taken together, these results demonstrate that the loss of DSTYK activates Wnt/β-catenin/LDHA signaling to promote the tumorigenesis of lung cancer and that DSTYK may be a therapeutic target.

## Introduction

Lung cancer is one of the most common malignant cancers [1, 2], and exploring its pathogenesis is of great significance for early diagnosis and treatment [3]. During the oncogenesis of lung cancer, many signals involved in embryonic development are activated, such as the *Wnt/β-catenin, TGF β*, and *Hedgehog* pathways [4–6]; an abnormal metabolism is one of the basic characteristics of tumor cells [7]. Therefore, investigating the regulatory mechanisms of these pathways and their effects on metabolism is vital for the design of intervention strategies.

The Wnt/β-catenin pathway plays an important role in the progression of lung cancer [8, 9]. A constitutively activating mutation of β-catenin was found in clinical specimens of lung cancer, which activated a signaling pathway in which β-catenin is the core element and resulted in tumorigenesis [10]. When cells are in a resting state, a degradation complex composed of *Axin, APC, GSK3β*, and *CK1α* within the cytoplasm phosphorylates β-catenin [11]. Phosphorylated β-catenin is bound by the E3 ligase β-TrCP, ubiquitinated and degraded through the proteasomal pathway. Phosphorylation of β-catenin at serine/threonine residues *33, 37, 41*, and *45* of its N-terminal domain is the core event in β-catenin regulation [12]. Currently, GSK3β and CK1α are thought to be responsible for the phosphorylation of these sites during individual embryonic development. CK1α phosphorylates serine residue *45* before GSK3β phosphorylates residues *33, 37*, and *41* [13]. The novel kinases that phosphorylate these amino acids in the context of tumor progression remain undetermined.

Dual serine/threonine and tyrosine protein kinase (DSTYK) encodes a protein product consisting of 850-931 amino acids [14]. The N-terminal domain of DSTYK is a noncatalytic domain with low conservation among species [14]. In contrast, its C-terminus is the kinase catalytic domain and shows high conservation among species [14]. DSTYK is expressed in the brain, heart, muscles, kidneys and lungs and is distributed in the cytoplasm and nucleus [14].

The deletion of DSTYK in zebrafish via positional cloning was found by CRISPR-Cas 9 screening to cause notochord development defects [15, 16]. Moreover, in a study of its molecular mechanisms, DSTYK was found to be necessary for lysosome synthesis in mammalian cells, promoting fusion of the late endosome and lysosomal system [15, 16]. Furthermore, DSTYK inhibited *mTORC1*, accelerated *TFEB* entry into the nucleus, and activated the expression of genes related to lysosome synthesis [15, 16].

Whole-exon sequencing showed that DSTYK exhibits multiple mutations in a variety of pathological conditions. DSTYK gene mutations have also been observed in unilateral renal agenesis [17]. In addition, DSTYK mutation is closely related to urinary tract deformity [18, 19]. A water maze experiment demonstrated that deletion of the DSTYK kinase domain seriously affected the learning capabilities and memory functions of mice [20]. Furthermore, a mutated DSTYK (*Met296Ile*) in intracranial solitary fibrous tumors promoted tumor metastasis by activating the ERK pathway [21]. To date, the function and mechanism of DSTYK in tumorigenesis remain unclear.

In this study, the expression pattern of DSTYK in lung adenocarcinoma was detected and the function and expression pattern of DSTYK in lung cancer were evaluated. Moreover, the biological function mechanism of DSTYK was investigated.

## Materials and methods

### Cell culture and transfection

Normal bronchial epithelial cell line Bease-2B and lung cancer cell lines were obtained from the Cell Bank of the Chinese Academy of Science (Shanghai, China) and maintained in DMEM (Gibco) containing 10% FBS (Gibco) and antibiotics in a 5% CO_2_ incubator. Cells were transfected with Lipofectamine 8000 (Invitrogen) according to manufacturer instructions. Stable cell lines were selected using puromycin (Sangon Biotechnology, A610593) for one week. Cell lines were authenticated using STR profile analysis and used within 3–20 passages of thawing the original stocks.

### qPCR

Total RNA was extracted from cells or tissues using TRIzol and reverse transcribed to cDNA using a kit (Takara). RT-PCR analysis was performed using an Applied Biosystems Prism 7900 HT sequence detection system with Universal SYBR qPCR Master Mix (Vazyme, China). The expression levels of each mRNA relative to the β-actin expression level were calculated based on the 2^-ΔΔCt^ method. The primer sequences were as follows: F, 5’-ttgcatactgatcctcgg-3’; R, 5’-tgtgcactagttcatact-3’.

### Immunohistochemistry (IHC)

30 clinical samples were collected from Shanghai Chest Hospital after the informed consent was obtained was obtained from all subject. This study was approved by the ethical committee of the Shanghai Chest Hospital. The clinical information was listed in Table S1. Tissues were embedded in paraffin and cut into 5 µm sections. Dewaxing and rehydration were performed by placing slides in a decreasing gradient series of xylol and ethanol. Antigen recovery was performed by incubating the sections in a pH 6.0 citrate sodium solution at 100 °C for 20 min. After blocking endogenous peroxidase activity using a kit (ZSGB-BIO, PV-8000), the tissues were incubated with primary antibody (anti-DSTYK, 1;100, Proteintech, 20102-1-AP; anti-β-catenin, 1;100, CST, 8480; or anti-LDHA, 1;100, Proteintech, 19987-1-AP) at 4 °C overnight. Then, secondary antibody was added, and the tissues were incubated at room temperature for 1 h. The signals were developed using DAB (ZSGB-Bio, Beijing, China), and the sections were stained with hematoxylin. Tissues were examined under a microscope, and scoring was performed as described.

### KP mouse model

*p53*^*f/f*^*; LSL-Kras*^*G12D*^ (KP) model C57BL/6 mice were housed under standard conditions with 12 h dark and 12 h light cycles.

To study the roles of DSTYK in the tumorigenesis in vivo, the sg con or sg DSTYK were inserted into the cassette of pSECC lentivirus vector which expressed cas 9 enzyme and Cre recombinase. To induce lung cancer, the 8-week-old male KP mice were randomly divided into two groups (5 mice in each group) and anesthetized with 2.5% avertin; full anesthetization and absence of reaction to pain were ensured. A total of 1 × 10^9^ viruses (OBIO, Shanghai) were administered per mouse (8 weeks old) using an intranasal/orthotropic infection protocol as described [22]. The administration of pSECC/Sg con viruses led to the deletion of P53 and activation of Kras^G12D^. The administration of pSECC/Sg DSTYK viruses led to the deletion of P53 and DSTYK and activation of Kras^G12D^. Twelve weeks later, the lungs were harvested, and western blot assays were performed.

For the examination of DSTYK protein levels in the KP tumors, the mice were treated with/without Ad-Cre virus (adenovirus). This study was approved by the ethical committee of the Shanghai Chest Hospital, and complied with the ethical regulations of ethical committee of the Shanghai Chest Hospital.

### Western blot

Cells were harvested with RIPA buffer. The protein concentration was determined using BCA. Then, SDS-PAGE was performed, and the proteins were transferred to a PVDF membrane. After blocking with 5% BSA at room temperature for 1 h, the membrane was incubated with the primary antibody for 4 h and sequentially with the secondary antibody for 1 h. Then, the signals were examined using an ECL kit. The information about the antibodies were: Anti-DSTYK (Proteintech, 20102-1-AP), anti-GAPDH (Santa Cruz, sc-47724), ant-β-catenin (CST, 8480), anti-phosphorylated β-catenin (CST, 9561), anti-GST (Santa Cruz, sc-138), anti-Flag (Proteintech, 80010-1-RR), anti-Cyclin D1 (Santa Cruz, sc-8396), anti-c-Jun (Santa Cruz, sc-74543), anti-c-Myc (Santa Cruz, sc-40), anti-LDHA (Proteintech, 19987-1-AP), anti-Tubulin (Santa Cruz, sc-166729).

### MTT assay

An MTT (Merck, 11465007001) kit was used. Cells were plated in 96-well plates at a density of 1000 cells per well, and each well was loaded with 200 µl of medium. On days 1, 3, 5, and 7, 20 µl of MTT solution were added to each well, and the plates were incubated for 4 h. Then, supernatants were removed, precipitates were dissolved in 200 µl of DMSO, and absorbance was measured.

### Colony formation assay

Cells were suspended in 2× DMEM at a density of 10^6^ cells/L. A bottom agar layer was prepared with an equal volume of 1.2% agar and 2× DMEM containing antibiotics and 20% FBS, and 3 ml of the mixture was added to a 6 cm dish. Thirty minutes later, a bottom agar layer was prepared with an equal volume of 1.2% agar and 2× DMEM with an additional 0.2 ml of the cell suspension. After softly mixing, the mixture was added to the dish with the bottom agar layer. Fourteen days later, colonies were counted.

### Sphere formation assay

Cells from each group were collected and digested into single cells. Subsequently, for each group, 2 × 10^4^ viable cells per well were counted and seeded in low-adherent 6-well plates (Corning, USA) in serum-free F12/MEM containing B27, EGF (20 ng/ml) and FGF (40 ng/ml). After incubation at 37 °C for 14 days, pictures were taken under a microscope, and tumor spheres in five separate fields were counted. EGF (R&D, 236-EG), FGF (R&D, 233-FB), and B27 (R&D, AR008) were obtained from R&D.

### Immunoprecipitation

Cells were scraped and proteins were extracted from the cells with 1 ml RIPA buffer. After centrifugation (4 °C, 12,000 rpm) for 20 min, supernatant was divided into two tubes and immunoprecipitated with the indicated antibody and IgG as control at 4 °C. On the next day, protein A beads (Sangon, Shanghai) were added to the supernatant and incubated for another 4 h. Then, the beads were collected through centrifugation and washed three times. After as much wash buffer as possible was removed, the beads were mixed with 30 µl of loading buffer and boiled for 5 min at 100 °C. The precipitates were examined using western blot.

### GST pull-down assay

Cells were scraped, and proteins were extracted from the cells with 1 ml RIPA buffer. After centrifugation (4 °C, 12,000 rpm) for 20 min, the supernatants were divided into two tubes and immunoprecipitated with 5 µg of GST or GST-DSTYK fusion protein at 4 °C. The next day, Sepharose 4B beads (GE Healthcare) were added to the supernatants and incubated for another 4 h. Then, the beads were collected through centrifugation and washed three times. After as much wash buffer as possible was removed, the beads were mixed with 30 µl of loading buffer and boiled for 5 min at 100 °C. The precipitates were then examined using western blot.

### Reporter assay

Cells at 70% confluence were plated in a 24-well plate. For each well, the cells were transfected with 0.01 µg of TOPFlash, 0.01 µg of TK Renilla, and 0.25 µg of DSTYK expression vector or empty vector. Forty-eight hours later, the cells were lysed with RIPA buffer, and reporter activity was examined using a dual reporter assay kit (Promega).

### In vitro kinase assay

Fusion protein GST-β-catenin (N) (the N-terminal domain of β-catenin) was purified and incubated with Flag-DSTYK protein immunoprecipitated from a stable A549 cell line in the presence of kinase assay buffer and ATP (CST, 9802). The reaction was performed at 32 °C for 30 min and stopped by the addition of loading buffer. The phosphorylation of β-catenin was examined using western blot.

### ChIP

ChIP was performed using a kit (Cell Signaling Technology, 9004) according to manufacturer instructions. The primers for qPCR were as follows: F, 5’-TCAAAACCAAGAAACTCAG-3’; F, 5’-GGGAAAGACCAAATGATTA-3’. qPCR was performed using a 2× SYBR qPCR mixture according to manufacturer instructions.

### Lactate measurement

A lactate assay was performed using a kit (Abcam, ab65331). Briefly, cell culture medium was collected. The culture medium and standards were added to the wells of a plate, after which reaction mixture was added and incubated for 30 min at room temperature. The samples were analyzed with a microplate reader according to manufacturer instructions.

### RNA sequencing

For RNA sequencing, A549 control cells and cells with the knockdown of DSTYK were harvested. The libraries were performed by Novogene (Tianjin, China), and an Illumina HiSeqX10 (Illumina, San Diego, CA, USA) was used for high-throughput sequencing. Quality control and removal of adapters of raw paired-end reads were performed using fastp (version 0.20.1). The gene expression counts were analyzed using HTseq-count software. The differently expressed genes were identified using DEseq2 (version 3.12) in R environment (version 3.6.3). Data were uploaded, and the GEO number is GSE178487.

### Statistical analysis

All experiments in this study were performed in triplicate and error bars represent the standard deviation (±S.D) of triplicate samples. Statistical analysis was conducted using GraphPad Prism (version 7.0). Comparisons between groups were performed using two-tailed independent sample Student’s *t*-tests analysis. Data were expressed as mean ± S.D, *P* < 0.05 was considered a significant difference (*P < 0.05; **P < 0.01; ***P < 0.001).

## Results

### Low DSTYK expression was observed in lung cancer

**Fig. 1:**
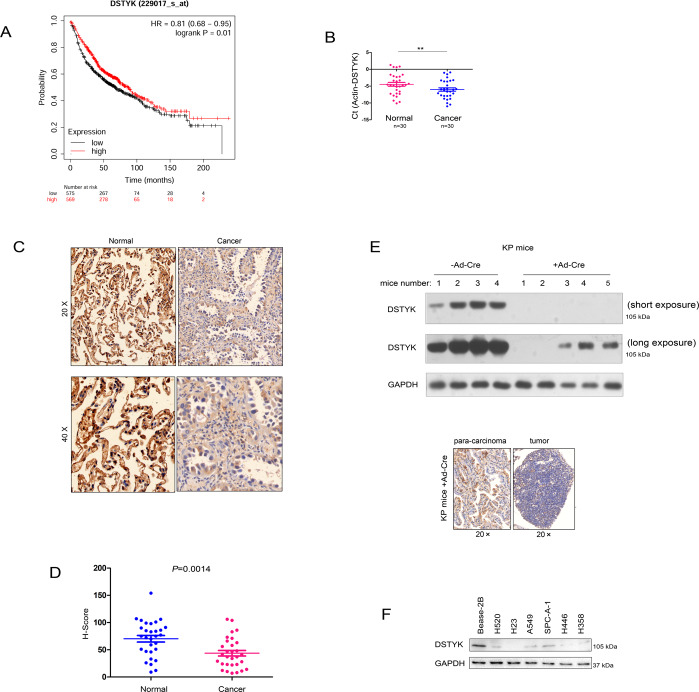
DSTYK was downregulated in lung cancer. **A** The KmPlot database was mined to confirm the correlation between the expression of DSTYK and the survival of patients with lung cancer. **B** qPCR was performed to determine the mRNA levels of DSTYK in lung cancer tissues and noncancerous tissues. **P < 0.01. **C** Immunohistochemistry (IHC) was performed to determine the protein levels of DSTYK in lung cancer tissues and noncancerous tissues. The images with different magnification were shown and the scale bar was indicated. **D** Statistical analysis of the data in **C**. The IHC intensity was scored and analyzed. **E** Western blot and IHC were performed to determine the protein levels of DSTYK in the KP mouse model. Eight-week-old KP mice in the experimental group were treated with Ad-Cre virus (+Ad-Cre) (10^9^ per mouse) to delete the expression of P53 and activate the expression of Kras^G12D^. Mice in the control group were treated with control virus. Twelve weeks later, the mice were killed, lung tissues were collected, and DSTYK protein levels were examined by western blot (up) and IHC (below). The images of western blot with long exposure and short exposure were shown. **F** The protein levels of DSTYK in normal lung epithelial cells (Bease-2B) and lung cancer cells.

To clarify the expression pattern of DSTYK in lung cancer, we initially searched the Kmplot database for correlations between DSTYK expression and lung cancer patient survival. Bioinformatics analysis showed that DSTYK expression in lung cancer was positively correlated with patient survival time (Fig. 1A). Next, we detected the mRNA and protein levels of DSTYK in 30 lung cancer tissues and 30 paracancerous tissues by qPCR. The mRNA level of DSTYK was low in lung cancer (Fig. 1B), and immunohistochemistry indicated that the protein level of DSTYK was low in lung cancer (Fig. 1C, D). The KP (*LSL-Kras*^*G12D*^*;P53* ^*loxp/loxp*^) mouse model is a classic model for investigating lung cancer. The western blot (Fig. 1E, up) and IHC (Fig. 1E, below) results showed that DSTYK expression levels was decreased in the tumorigenesis of KP mice (the mice were treated with Ad-Cre virus to induce the tumor). Finally, DSTYK expression in lung cancer cell lines was detected by Western blot. As shown in Fig. 1F, DSTYK was highly expressed in the normal pulmonary bronchial epithelial cell line *Bease-2B* (Fig. 1F). These results indicate that DSTYK expression is downregulated in lung cancer.

### DSTYK inhibited the growth, colony formation, and sphere formation of lung cancer cells

**Fig. 2:**
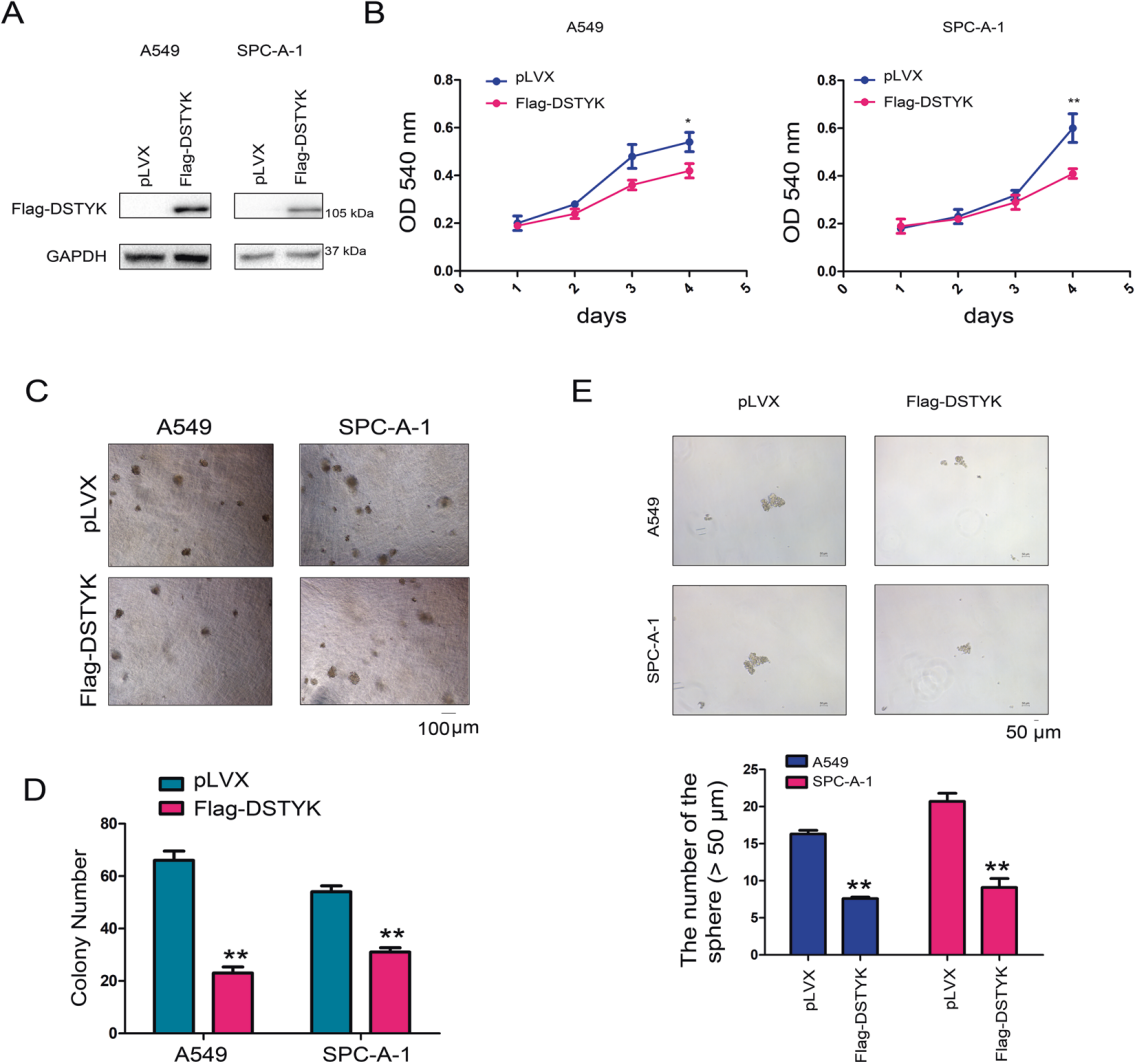
DSTYK inhibited the growth of A549 and SPC-A-1 cells. **A** Western blot was performed to examine the overexpression of DSTYK. A549 and SPC-A-1 cells were infected with lentivirus expressing Flag-tagged DSTYK and selected with puromycin. Then, western blotting was performed. **B** The MTT assay was performed to examine the effects of DSTYK on the growth of A549 and SPC-A-1 cells. **C, D** A colony formation assay was performed, and the results were quantified. **E** Sphere formation was assessed to examine the roles of DSTYK in stemness. Details about sphere formation are described in the “Materials and methods”. The spheres were photographed and counted. The scale bar was indicated. *P < 0.05; **P < 0.01.

**Fig. 3:**
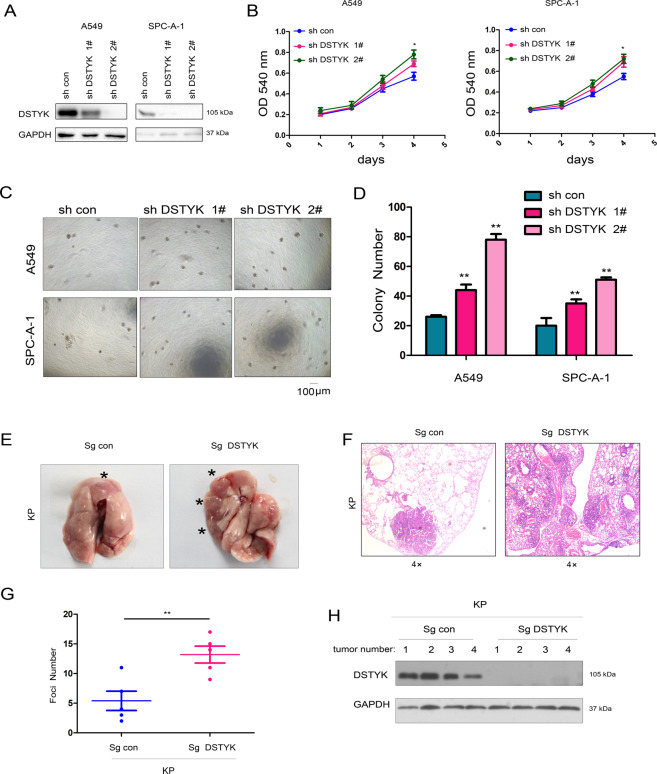
Knockdown of DSTYK promoted the growth of A549 and SPC-A-1 cells. **A** Western blot was performed to examine the knockdown efficiency. A549 and SPC-A-1 cells were infected with lentivirus expressing shRNA for DSTYK for 24 hours and selected with puromycin. Then, western blotting was performed. **B** The MTT assay was performed to examine the effects of DSTYK knockdown on the growth of A549 and SPC-A-1 cells. **C, D** A colony formation assay was performed, and the results were quantified using control cells and DSTYK-knockdown cells. **E–H** A tumorigenesis assay was performed to determine the functions of DSTYK in lung cancer. To induce lung cancer, the 8-week-old male KP mice were randomly divided into two groups (5 mice in each group) and anesthetized with 2.5% avertin; full anesthetization and absence of reaction to pain were ensured. A total of 1 × 10^9^ viruses (pSECC/Sg con or pSECC/Sg DSTYK) (OBIO, Shanghai) were administered per mouse using an intranasal/orthotropic infection protocol. The administration of pSECC/Sg con viruses led to the deletion of P53 and activation of Kras^G12D^. The administration of pSECC/Sg DSTYK viruses led to the deletion of P53 and DSTYK and activation of Kras^G12D^. Twelve weeks later, the mice were killed, lung tissues were collected and photographed, and the tumors were indicated with “*” (**E**), the tumors were examined with HE staining and analyzed (**F, G**), and the expression of DSTYK in the tumors was examined using western blot. The scale bar was indicated. *P < 0.05; **P < 0.01.

Downregulated DSTYK expression in lung cancer implies that DSTYK plays an important role in lung cancer progression. We overexpressed DSTYK in A549 and SPC-A-1 cells to further explore the function of DSTYK in the progression of lung cancer (Fig. 2A). Figure 2B shows that DSTYK overexpression inhibited the growth of lung cancer cells in liquid medium. Anchorage-independent growth is a basic characteristic of tumor cells. Therefore, we evaluated the effect of DSTYK expression on the anchorage-independent growth of lung adenocarcinoma cells via soft agar colony formation and sphere formation assays. The results showed that DSTYK overexpression inhibited colony formation on soft agar (Fig. 2C, D) and sphere-forming abilities of lung cancer cells (Fig. 2E). However, when DSTYK expression was knocked down in A549 and SPC-A-1 cells (Fig. 3A), various functional tests indicated that DSTYK knockdown promoted growth (Fig. 3B) and colony formation (Fig. 3C, D) of A549 and SPC-A-1 cells. Moreover, knockout of the expression of DSTYK in the KP mouse model promoted tumorigenesis (Fig. 3E-H). These results confirm that DSTYK inhibits the progression of lung tumors.

### Interaction between DSTYK and β–catenin in lung cancer cells

**Fig. 4:**
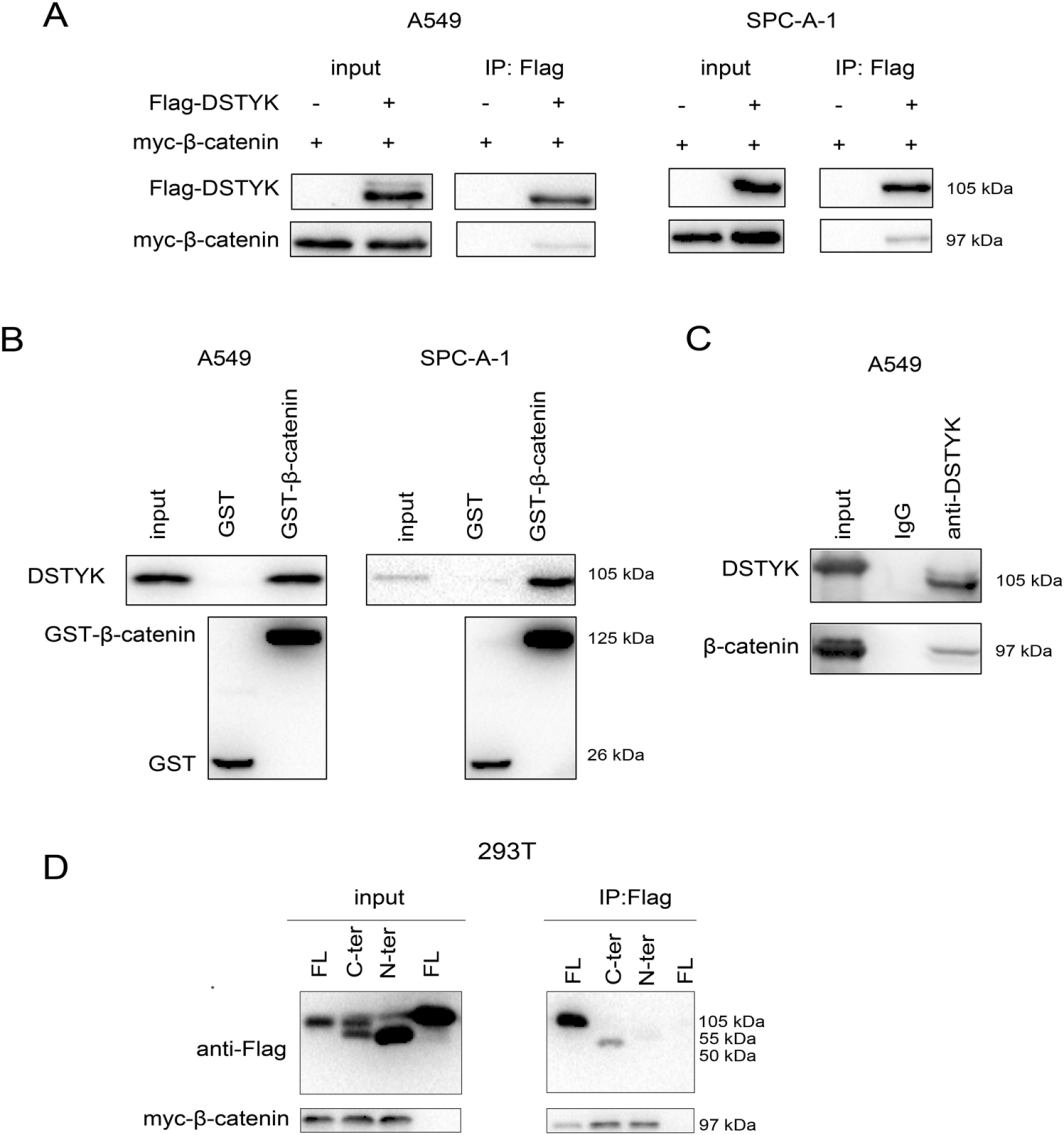
DSTYK interacts with β-catenin. **A** The interaction between exogenously expressed Flag-DSTYK and myc-β-catenin was examined using immunoprecipitation. Cells were harvested 48 h after transfection. **B** A GST pull-down assay was performed to confirm the interaction between endogenous β-catenin and the fusion protein GST-DSTYK. **C** The interaction between exogenously expressed Flag-DSTYK and myc-β-catenin was examined using immunoprecipitation. Cells were harvested and lysed with RIPA buffer, and anti-β-catenin antibody was added for immunoprecipitation. **D** An immunoprecipitation assay was performed to determine the domain of DSTYK that binds myc-β-catenin. N-ter the N-terminus of DSTYK (1–500 aa), C-ter the C-terminus of DSTYK (501–929 aa), FL the full length of DSTYK.

We detected the interactions among DSTYK and key components of the Wnt/β-catenin and TGFβ signaling pathways to explore the molecular mechanism by which DSTYK regulates lung cancer and found an interaction between exogenous DSTYK expression and β-catenin (Fig. 4A). A GST pull-down test showed the same result (Fig. 4B). Moreover, endogenously expressed DSTYK in lung adenocarcinoma cells interacted with β-catenin (Fig. 4C), and an interaction between the N-terminal domain of DSTYK and β-catenin was observed by immunoprecipitation (Fig. 4D).

### DSTYK phosphorylated β-catenin and inhibited the Wnt/β-catenin signaling pathway

**Fig. 5:**
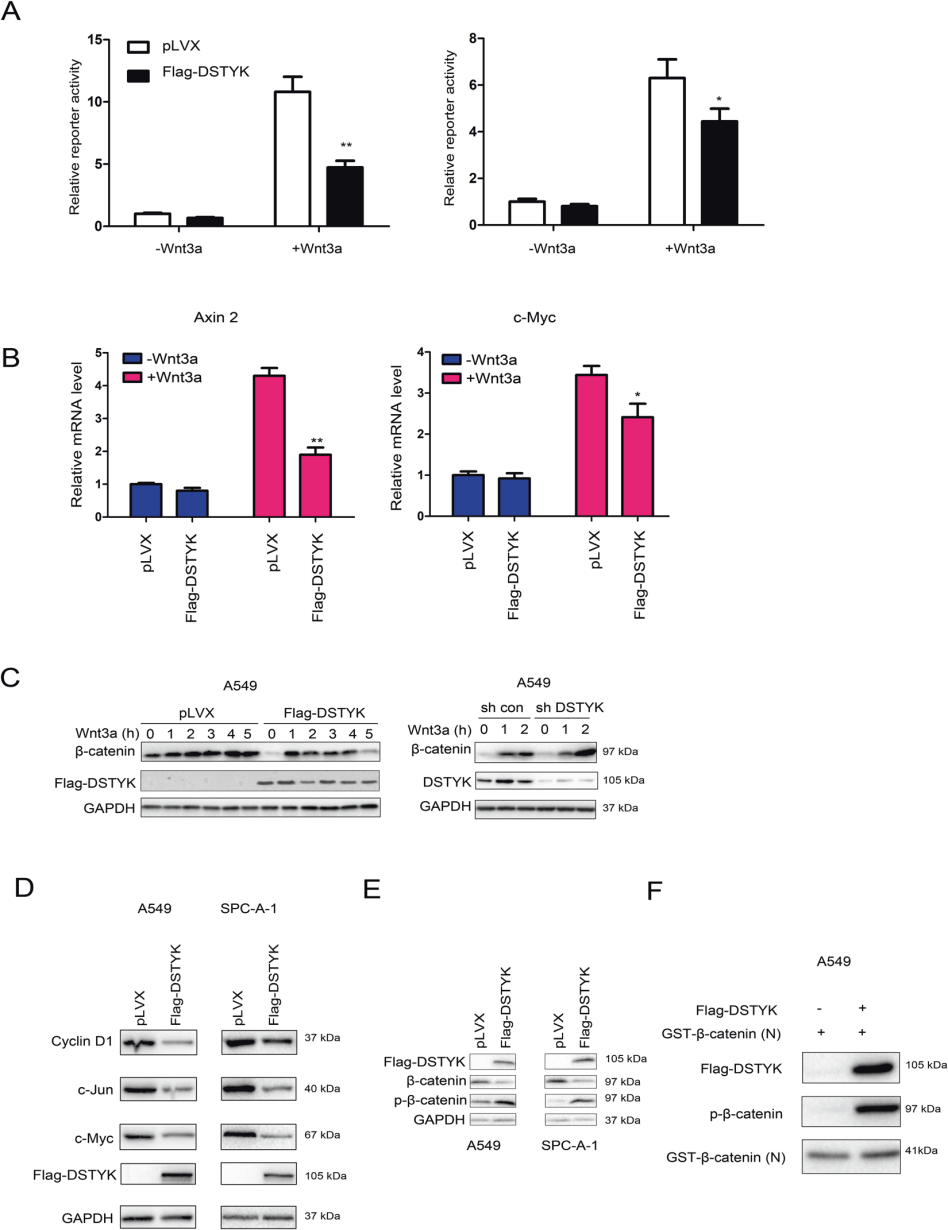
DSTYK inhibited Wnt/β-catenin signaling and promoted the phosphorylation of β-catenin. **A** The TOPFlash reporter assay was performed to examine the effects of DSTYK on the transcriptional activity of β-catenin. **B** qPCR was performed to examine the effects of DSTYK on the expression of Axin2 and c-Myc. **C** Western blot was performed to examine the effects of DSTYK on the accumulation of β-catenin. Control cells and DSTYK-overexpressing cells were treated with Wnt3a for the indicated duration, and the protein levels of β-catenin were examined. **D** Western blot was performed to examine the effects of DSTYK on the expression of cyclin D1 and c-Myc. **E** The phosphorylation of β-catenin was examined after overexpression of DSTYK. F. In vitro kinase assay. Flag-DSTYK was immunoprecipitated and incubated with GST-N-β-catenin at 32 °C for 30 min, and the phosphorylation of β-catenin was examined. *P < 0.05; **P < 0.01.

We first investigated the effect of DSTYK expression on the TOPFlash reporter to elucidate the influence of DSTYK on the Wnt/β-catenin signaling pathway. The results showed that DSTYK overexpression inhibited the Wnt3a-induced activation of the TOPFlash reporter (Fig. 5A), the mRNA levels of Axin2 and c-Myc (two targets of β-catenin) (Fig. 5B) and the accumulation of β-catenin (Fig. 5C). Similarly, DSTYK overexpression inhibited the expression of target genes downstream of β-catenin (Fig. 5D). Moreover, DSTYK overexpression elevated the phosphorylation level of β-catenin (Fig. 5E). Because DSTYK has serine/threonine kinase activity and interacts with β-catenin, we conducted an in vitro kinase reaction to detect the phosphorylation effect of DSTYK on β-catenin. The results indicate that DSTYK phosphorylated β-catenin in vitro (Fig. 5F).

### LDHA is a target gene of the Wnt/β-catenin signaling pathway

**Fig. 6:**
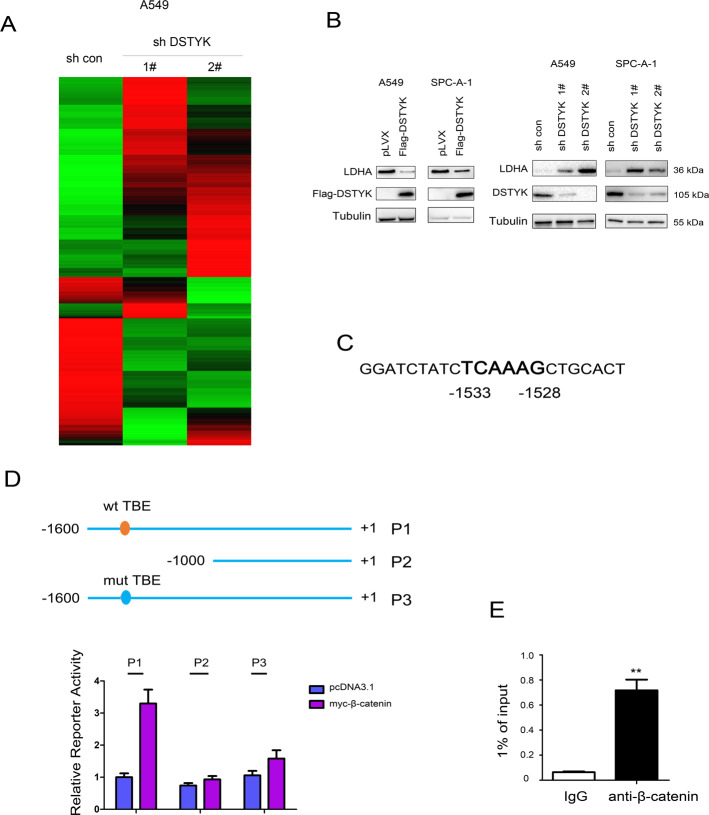
β-Catenin regulated the expression of LDHA. **A** The heatmap of the RNA-seq was shown. **B** The protein levels of LDHA in cells with DSTYK overexpression or knockdown. **C** The sequence of the human LDHA promoter. **D** The TBE sequence at −1533 to −1528 in the LDHA promoter was indicated and mutated for the luciferase assay. **E** ChIP was performed to examine the binding of β-catenin to the LDHA promoter. **P < 0.01.

Next, the RNA-seq was performed using A549 control cells and A549 cells with DSTYK knockdown, which suggested that knockdown of DSTYK upregulated the mRNA levels of LDHA (Fig. 6A, The GEO number is GSE178487). Therefore, we next verified the results of RNA-seq. It was found that the LDHA protein level was inhibited by DSTYK overexpression and upregulated by knockdown of DSTYK expression (Fig. 6B). Moreover, we found a binding site for β-catenin/TCF (TBE, TCF-binding element) in the LDHA promoter region and analyzed the LDHA promoter (Fig. 6C). After TBE was removed or mutated, β-catenin had no significant activation effect on the LDHA promoter (Fig. 6D). In addition, chromatin immunoprecipitation showed that β-catenin formed a complex with the LDHA promoter (Fig. 6E). These results suggest that LDHA is a target gene of the Wnt/β-catenin signaling pathway.

### DSTYK exerts biological functions through Wnt/β-Catenin/LDHA signaling

**Fig. 7:**
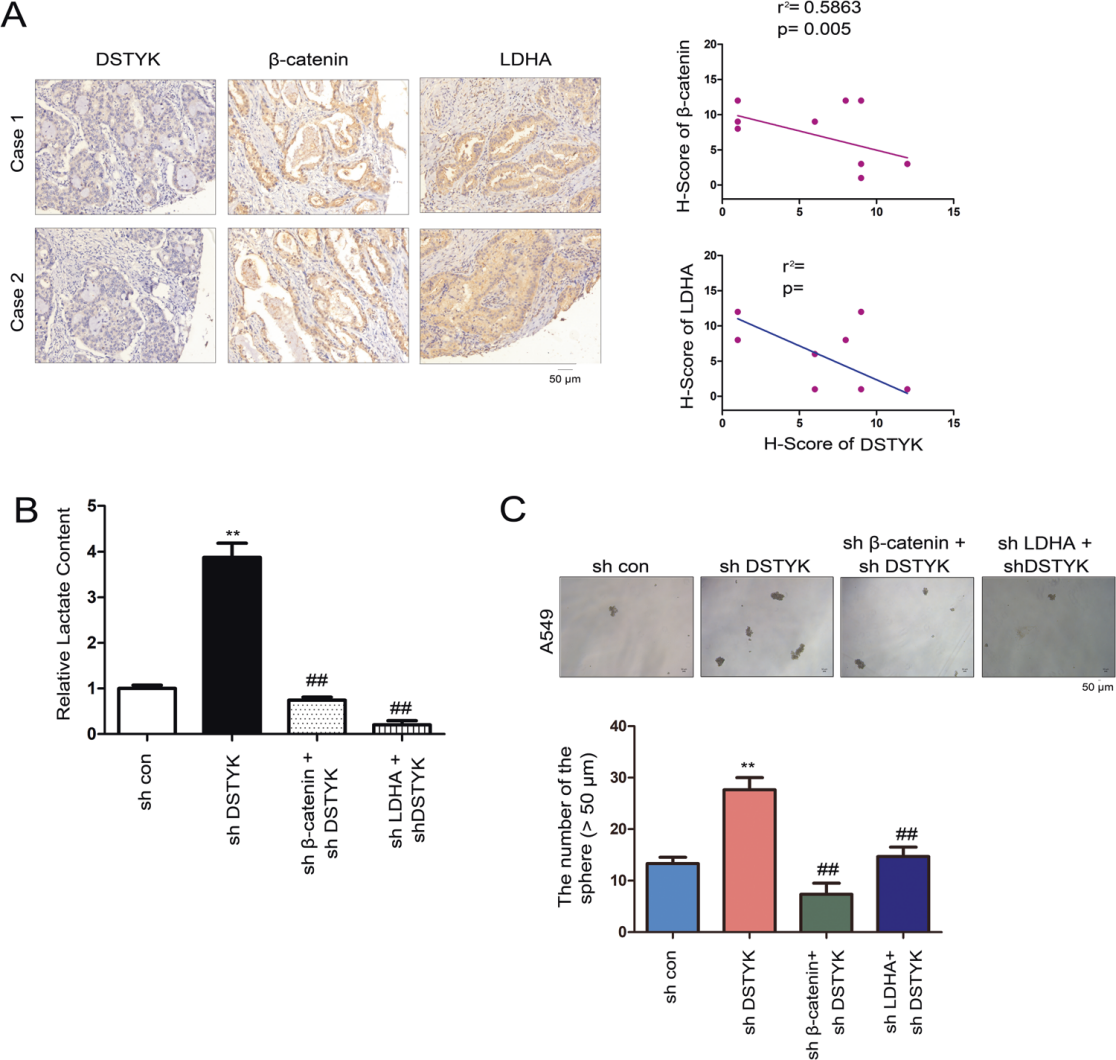
DSTYK/Wnt/β-catenin signaling regulated LDHA expression. **A** The correlation between DSTYK, β-catenin and LDHA in clinical lung tissues. The protein levels of DSTYK, β-catenin and LDHA in 10 lung cancer tissues were examined. The immunostaining was scored. Representative images of two cases are shown, and the correlations between the expression of DSTYK, β-catenin, and LDHA were analyzed. **B** Knockdown of β-catenin or LDHA abolished the elevated lactate content induced by DSTYK knockdown. ^##^*P* < 0.01; **P < 0.01. **C** Knockdown of β-catenin or LDHA abolished the enhanced sphere formation induced by DSTYK knockdown. The sphere was counted and the statistical analysis was performed. **P < 0.01; ^##^*P* < 0.01. The scale bar was indicated.

We analyzed whether the biological function of DSTYK depends on the Wnt/β-catenin/LDHA signaling pathway. In clinical lung cancer specimens, DSTYK protein level was inversely correlated with the expression of β-catenin and LDHA (Fig. 7A). Moreover, the lactate content was upregulated in lung cancer cells in which DSTYK expression was disturbed; this upregulation could be restored by knockdown of β-catenin or LDHA expression (Fig. 7B). In terms of biological function, disturbing DSTYK expression promoted sphere formation, which could be restored in the manner stated above (Fig. 7C). These results indicate that the biological effects of DSTYK occur via Wnt/β-catenin/LDHA signaling.

## Discussion

In this study, we found that DSTYK expression was downregulated in lung adenocarcinoma. Upregulation of DSTYK expression in lung adenocarcinoma inhibited the growth of cancer cells, colony formation, and sphere formation; downregulation of DSTYK expression accelerated lung cancer cell growth and colony formation. Additionally, knockout of DSTYK expression in the KP mouse model of lung adenocarcinoma promoted the development of lung cancer. An experiment on the molecular mechanism showed that DSTYK upregulated the level of β-catenin phosphorylation and inhibited the Wnt/β-catenin signaling pathway. Lactate dehydrogenase (LDHA) was found to be a downstream target gene of the DSTYK/Wnt/β-catenin signaling pathway; thus, the biological function of DSTYK was determined to be exerted through the activation of Wnt/β-catenin/LDHA signaling. This study reveals the function and mechanism of DSTYK in lung cancer and suggests the importance of DSTYK agonists in the treatment of lung cancer.

The phosphorylation of β-catenin by DSTYK is one of the most novel findings in this study. Kinases are important targets for tumor therapy. Previous studies have shown that GSK3β and CK1α are the most important kinases in the Wnt/β-catenin signaling pathway during embryonic development [13]. The biological context of tumorigenesis and progression completely differs from that of embryonic development. Therefore, it is likely that novel kinases regulate the Wnt/β-catenin signaling pathway in tumorigenesis. This study also reveals phosphorylation of amino acid residues 33, 37, 41, and 45 of β–catenin, which contributes to further understanding of the Wnt/β-catenin signaling pathway.

The regulation of LDHA expression through the Wnt/β-catenin signaling pathway was also found in this study. LDHA catalyzes lactate production, and glycolysis is one of the basic characteristics of tumor cell metabolism [23, 24]. Upon producing lactate, tumor cells quickly obtain intermediate materials for ATP and biomacromolecule synthesis, reshaping the tumor microenvironment [23].

Multiple studies have demonstrated the roles of LDHA in the progression of lung cancer. The expression level of LDHA has been negatively correlated with the survival of lung cancer patients [25]. LDHA has also been shown to mediate the functions of many oncogenes in lung cancer [26]. Treatment of lung cancer cells with lactic acid promotes the migration and infiltration of lung cancer cells by upregulating the expression of snail and inhibits the senescence of lung cancer cells [27]. Moreover, lactic acid upregulates the expression of PD-L1 in lung cancer cells and remodels the immune microenvironment of tumor cells [28]. Consistently, LDHA inhibitor inhibits the tumorigenicity of A549 cells in nude mice [29]. In fact, several studies have revealed that tumor metabolism is regulated by the Wnt/β-catenin signaling pathway. Deng et al. found that the Wnt/β-catenin signaling pathway accelerated the progression of pancreatic cancer by directly activating the expression of enzymes (e.g., HMGCR and HMGCS) related to mevalonate metabolism [30]. Furthermore, some reports have shown that PDK1, a key enzyme for glucose metabolism, is a target gene of the Wnt/β-catenin signaling pathway in liver cancer [31]. These studies have demonstrated that the Wnt/β-catenin signaling pathway plays a significant role in the regulation of tumor metabolism.

In summary, this study revealed the expression pattern of DSTYK in lung cancer, clarified its function and mechanism in lung cancer, and provided a new target for the treatment of lung cancer.

## Supplementary information

The clinical information of the patients

## Data Availability

The datasets used and analyzed during the current study are available from the corresponding author on reasonable request.
